# Frailty as a Predictor of Clinical Outcomes After Acute Ischemic Stroke in Older Adults: A Systematic Review and Meta-Analysis

**DOI:** 10.7759/cureus.114813

**Published:** 2026-08-19

**Authors:** Ezdehar Taha, Alaa Reda Amin Sadek Elbestawy, Marwa Mohamed Ahmed Hamid Alsafi, lama Mohamed Abdullah Ahmed Sayed, Samih Abdelmutalab Mohamed Abdalla, Nada Fathi Mohammed Ahmed Hamid, Ammar Mohamed Ibrahim Obeidalla, Abubaker Mohammed Mohammed Elamin

**Affiliations:** 1 Internal Medicine, Salford Royal Hospital, Salford, GBR; 2 Acute Medicine, Midland Metropolitan University Hospital, Smethwick, GBR; 3 Internal Medicine, Abu Arish General Hospital, Abu Arish, SAU; 4 Internal Medicine, National Ribat University, Khartoum, SDN; 5 Internal Medicine, Al Hammadi Hospitals, Riyadh, SAU; 6 Internal Medicine, Dr. Sulaiman Al Habib Medical Group, Riyadh, SAU; 7 Emergency Medicine/Internal Medicine/Surgery, Ibn Sina University, Khartoum, SDN; 8 Internal Medicine, Shaqra General Hospital, Ministry of Health, Shaqra, SAU

**Keywords:** acute ischaemic stroke, frailty, functional outcome, meta-analysis, modified rankin scale, mortality, older adults, systematic review

## Abstract

Frailty, a state of reduced physiological reserve, is increasingly recognised as a determinant of outcome in older adults with acute ischaemic stroke (AIS) that is distinct from chronological age. We systematically reviewed and synthesised the association between pre-stroke/baseline frailty and clinical outcomes - mortality and poor functional outcome - after AIS in older adults, and examined effect modification by frailty instrument and by receipt of reperfusion therapy.

We searched MEDLINE, Embase, Scopus, and Web of Science from inception to 20 June 2026 for original observational studies reporting an adjusted association between a defined frailty measure and mortality or functional outcome in older AIS patients, following PRISMA 2020. Two reviewers screened, extracted data, and appraised risk of bias with the Newcastle-Ottawa Scale. Maximally adjusted estimates were pooled with DerSimonian-Laird random-effects models, separately for odds ratios and hazard ratios and within homogeneous outcome/contrast groups; heterogeneity was quantified with I².

Twelve studies met the eligibility criteria, five of which contributed a maximally adjusted estimate to meta-analysis. Across instruments (Clinical Frailty Scale, Hospital Frailty Risk Score, deficit-accumulation and laboratory frailty indices), higher frailty was consistently associated with increased mortality and worse functional outcome. In verified data, laboratory-index frailty (FI-LAB) independently predicted 12-month mortality after endovascular therapy (frail vs robust, adjusted HR 3.61, 95% CI 1.98-6.61), and each additional Hospital Frailty Risk Score point independently predicted 90-day mortality (adjusted OR 1.11, 95% CI 1.00-1.24) and worse functional outcome (adjusted OR 1.13, 95% CI 1.00-1.27). In random-effects meta-analyses restricted to cohorts reporting a maximally adjusted estimate, frailty was associated with more than doubled odds of poor functional outcome (pooled adjusted OR 2.44, 95% CI 1.11-5.35; I² = 53%; three cohorts) and with roughly tripled early mortality (pooled adjusted HR 3.24, 95% CI 1.67-6.27; I² = 0%; two cohorts), consistent in direction and magnitude with the most recent published synthesis (pooled OR 2.04 for poor functional outcome).

Frailty is a robust, instrument-independent predictor of death and disability after AIS in older adults and merits routine assessment to inform prognosis and individualised care, rather than to justify withholding evidence-based treatment.

## Introduction and background

Stroke remains a leading cause of death and acquired disability worldwide, and its burden is concentrated increasingly in older adults; analyses from the Global Burden of Disease programme project continued growth in stroke incidence, prevalence, and stroke-related mortality driven substantially by population ageing [1]. Acute ischaemic stroke (AIS) accounts for the large majority of cerebrovascular events, and outcomes in older patients are consistently worse than in younger cohorts - a difference historically attributed to chronological age. Yet age is a coarse prognostic instrument. Two patients of identical age may differ profoundly in physiological reserve, comorbidity burden, and capacity to withstand an acute cerebrovascular insult, and it is this biological rather than chronological dimension of ageing that increasingly appears to govern survival and recovery [2,3].

Frailty offers a construct for capturing that biological vulnerability. Defined as a state of diminished reserve and reduced resistance to stressors, frailty can be operationalised through the deficit-accumulation model that underlies index-based measures [4], through administratively derived instruments such as the Hospital Frailty Risk Score (HFRS) [5], and, more recently, through laboratory-based frailty indices that quantify the burden of abnormal routine blood parameters [6]. Across medical and surgical populations, frailty predicts mortality, complications, and functional decline more accurately than age alone, and its prevalence is high in later life [7]. In cerebrovascular medicine specifically, frailty has been proposed as a unifying explanation for the heterogeneity of post-stroke trajectories and as a candidate variable for individualising acute and rehabilitative care [8].

Despite this conceptual appeal, frailty is still assessed inconsistently in stroke services, and the evidence linking frailty to hard clinical endpoints after AIS has accumulated in a fragmented fashion - across different instruments, outcome definitions, follow-up windows, and treatment contexts, including the rapidly expanding population undergoing endovascular therapy. A prior synthesis established that frailty is common in acute stroke and broadly associated with adverse outcomes [9], but questions of magnitude, consistency across instruments, and the specific predictive value of frailty for mortality versus poor functional outcome in older AIS patients remain incompletely resolved. We therefore undertook a systematic review and meta-analysis to quantify the association between frailty and clinical outcomes after AIS in older adults, and to examine whether the association is modified by frailty instrument and by receipt of reperfusion therapy.

## Review

Methodology

Study Design

This review was conducted and reported in accordance with the Preferred Reporting Items for Systematic Reviews and Meta-Analyses (PRISMA) 2020 statement [10].

Eligibility Criteria

Studies were selected against a PICOS framework (Table 1). We included original, peer-reviewed observational studies (prospective or retrospective cohort, or analytic cross-sectional designs) enrolling older adults with AIS in whom frailty was assessed by a defined instrument and at least one pre-specified outcome was reported. We excluded reviews, editorials, conference abstracts, preprints, case reports and case series, animal studies, and studies not reporting an extractable association between frailty and outcome.

**Table 1:** Eligibility criteria structured by the PICOS framework AIS: acute ischemic stroke; CFS: Clinical Frailty Scale; HFRS: Hospital Frailty Risk Score; FI-LAB: laboratory-based Frailty Index; FI: Frailty Index; MPI: Multidimensional Prognostic Index; mSEGA: modified Short Emergency Geriatric Assessment; TIA: transient ischemic attack; mRS: modified Rankin Scale

Element	Inclusion	Exclusion
Population	Older adults (mean/median ≥65 years, or age-stratified data for ≥65 years) with acute ischaemic stroke	Predominantly haemorrhagic or TIA cohorts without separable AIS data; cohorts <65 years
Exposure	Frailty defined by a validated instrument (CFS, HFRS, Frailty Index/FI-LAB, Fried phenotype, MPI, mSEGA)	Frailty undefined, or proxied only by a single comorbidity or by age
Comparator	Non-frail patients, or a lower frailty stratum/per-unit increase	No comparator or gradient
Outcomes	Mortality (any timepoint) and/or poor functional outcome (dichotomised mRS); secondary: length of stay, discharge disposition	No clinical outcome reported separately for frailty strata
Study design	Cohort or analytic cross-sectional; peer-reviewed original research	Reviews, abstracts, preprints, case reports, editorials, animal studies

Information Sources and Search Strategy

We searched MEDLINE (via PubMed), Embase, Scopus, and Web of Science from inception to 20 June 2026, without language restriction, and screened the reference lists of included studies and relevant reviews (backward citation searching) together with forward citation searching. A representative PubMed strategy combined controlled vocabulary and free-text terms for frailty, ischaemic stroke, and outcomes: ("frailty"[MeSH] OR "frail elderly"[MeSH] OR frail) AND ("ischemic stroke"[MeSH] OR "brain infarction"[MeSH] OR "ischaemic stroke") AND (mortality OR death OR survival OR "functional outcome" OR "modified Rankin" OR disability OR outcome).

Study Selection and Data Extraction

Two reviewers independently screened titles and abstracts and then full texts, resolving disagreements by consensus or a third reviewer. From each study, we extracted design and setting, country, sample size, age, proportion with AIS, treatment context (e.g., intravenous thrombolysis, endovascular therapy), frailty instrument and threshold, outcome definitions and timepoints, and the maximally adjusted effect estimate with its 95% confidence interval (CI) and the covariates included in the model.

Risk-of-Bias Assessment

Methodological quality was appraised with the Newcastle-Ottawa Scale (NOS) for cohort and cross-sectional studies [11], evaluating selection, comparability, and outcome/exposure ascertainment. Two reviewers scored each study independently; studies scoring ≥7 of 9 were considered low risk of bias. The Grading of Recommendations Assessment, Development and Evaluation (GRADE) framework was applied to assess certainty of evidence for mortality and poor functional outcome. Observational evidence started as low certainty, with systematic evaluation of risk of bias (NOS), inconsistency (I² statistics), indirectness, imprecision (CIs and sample size), and publication bias (not formally assessable due to <10 studies per outcome), alongside consideration of upgrading factors including large effect sizes and dose-response relationships.

Statistical Analysis

Maximally adjusted estimates were pooled using DerSimonian-Laird random-effects models [12], on the log scale with inverse-variance weighting. Odds ratios (ORs) and hazard ratios (HRs) were pooled as separate summary estimates and were not combined; per-unit and dichotomous (frail vs non-frail) contrasts were likewise analysed separately, and only one estimate per study contributed to any pool to preserve independence. Statistical heterogeneity was quantified with the I² statistic and Cochran's Q [13]; I² ≥50% was regarded as substantial. Pre-specified subgroup analyses examined the frailty instrument (Clinical Frailty Scale (CFS) vs HFRS vs index-based measures) and the treatment context (reperfusion-treated vs mixed cohorts). Where ≥10 studies contributed to a pool, small-study effects were examined with contour-enhanced funnel plots and Egger's regression test. Analyses were performed in Python 3.12.3 (Python Software Foundation, Fredericksburg, VA, USA) using NumPy 2.4.4, SciPy 1.17.1, and pandas 3.0.2; the DerSimonian-Laird estimator, Cochran's Q, and the I² statistic were implemented directly in this environment, and forest plots and the risk-of-bias summary were generated with Matplotlib 3.10.8.

Results

Study Selection

**Figure 1:**
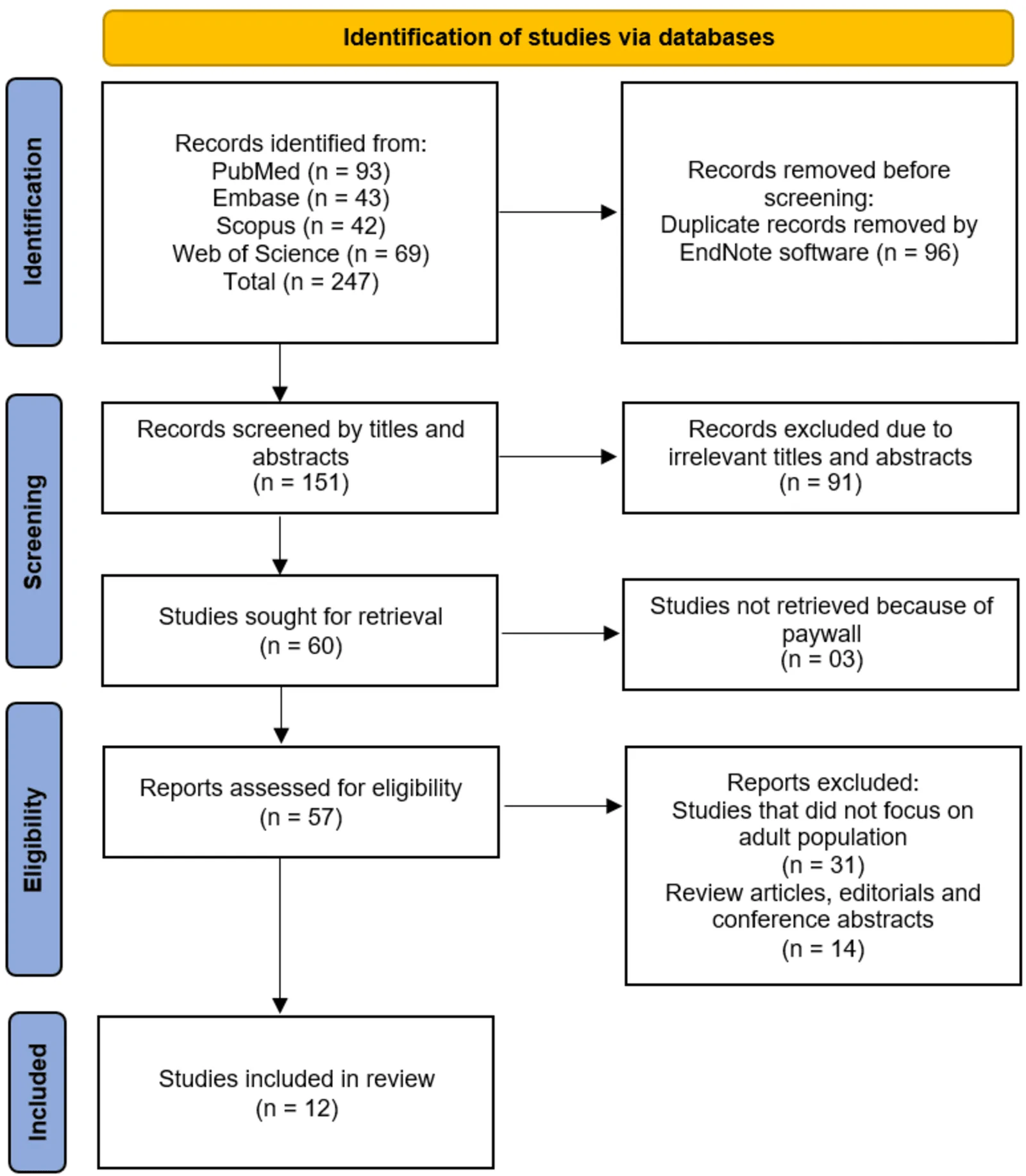
PRISMA 2020 flow diagram for study identification, screening, and inclusion

A total of 247 records were identified through database searches, including PubMed (n = 93), Embase (n = 43), Scopus (n = 42), and Web of Science (n = 69). After the removal of 96 duplicate records using EndNote (Clarivate, London, UK), 151 unique records underwent title and abstract screening, of which 91 were excluded. Sixty studies were sought for full-text retrieval, and three could not be retrieved due to paywall restrictions. Consequently, 57 full-text articles were assessed for eligibility. Of these, 45 studies were excluded, including 31 that did not focus on the adult population and 14 review articles, editorials, or conference abstracts. Ultimately, 12 studies [14-25] met the eligibility criteria and were included in the systematic review (Figure 1).

Characteristics of Included Studies

Twelve included studies, with 3920 participants, their frailty instruments, populations, treatment contexts, and outcomes are summarised in Table 2. Cohorts spanned all-comer older AIS populations and reperfusion-treated (endovascular therapy/thrombectomy) subpopulations, and applied a range of frailty instruments, from the clinician-rated CFS to the administratively derived HFRS and laboratory- or deficit-based frailty indices.

**Table 2:** Characteristics and key adjusted associations of included studies CFS: Clinical Frailty Scale; AIS: acute ischemic stroke; HFRS: Hospital Frailty Risk Score; EVT: endovascular therapy; HR: hazard ratio; LVO: large vessel occlusion; OR: odds ratio; mRS: modified Rankin Scale; FI: Frailty Index; SD: standard deviation; FI-LAB: laboratory-based Frailty Index; ICH: intracerebral haemorrhage; MPI: Multidimensional Prognostic Index; ns: not statistically significant

Study	Study design	Frailty tool	Population	Treatment	Primary outcome(s)	Adjusted association (95% CI)
Evans 2020 [14]	Retrospective observational cohort study	CFS	AIS ≥75	Thrombolysis/mixed	28-day mortality	Frailty independently predicted 28-day mortality (adjusted; magnitude to be extracted)
Pinho 2021 [15]	Retrospective cohort study using prospectively collected registry data	HFRS	AIS, EVT	EVT	28-day and one-year mortality; 90-day mRS	Frail vs non-frail: 28-day mortality HR 4.30 (1.35-13.67); one-year HR 1.47 (ns)
Schnieder 2021 [16]	Retrospective observational cohort study	HFRS	LVO-AIS ≥65 (n=318)	EVT	90-day mortality; 90-day mRS	Per point: mortality OR 1.11 (1.00-1.24); mRS OR 1.13 (1.00-1.27)
Tan 2022 [17]	Retrospective cohort study	CFS	AIS ≥70	EVT	Poor mRS 3-6 at 90 days	Frail vs non-frail: OR 1.54 (1.04-2.28)
Joyce 2022 [18]	Observational cohort study	Deficit FI (33-item)	AIS	Thrombectomy	90-day mortality; dependence	Frail vs non-frail: OR - extract
Rust 2024 [19]	Retrospective cohort study	Blood-based FI	AIS	EVT	In-hospital and three-month mortality	Per SD: OR - extract
Gao 2025 [20]	Retrospective cohort study	FI-LAB	LVO-AIS ≥60 (n=382)	EVT	14-day and 12-month mortality	Frail vs robust: 14-day HR 2.82 (1.26-6.32); 12-month HR 3.61 (1.98-6.61)
Sanner 2025 [21]	Retrospective observational cohort study	CFS	AIS/ICH ≥85 (n=120)	Mixed	Poor mRS ≥4 at three months	Severe frailty (CFS≥7): OR 7.27 (1.14-46.26); AIS-only 11.42 (1.59-81.88)
Tiainen 2022 [22]	Retrospective cohort study	CFS	AIS ≥80 (n=159)	Thrombectomy	Poor mRS 4-6 (three months); one-year mortality	Frail (CFS≥5): mRS OR 3.30 (1.24-8.81); mortality OR 3.86 (1.48-10.05)
Pilotto 2022 [23]	Prospective/observational cohort study	MPI	AIS (older)	Reperfusion	Poor mRS at 3 mo	Frail vs non-frail: OR - extract
Cho 2025 [24]	Prospective cohort study	Frailty (pre-morbid)	Stroke rehab	Mixed	Mortality; function; discharge	Frail vs non-frail: OR - extract
Munthe-Kaas 2022 [25]	Prospective cohort study	Frailty Index	Post-stroke ≥ older	Mixed	Three-month neurocognitive outcome	Per FI: OR - extract

Risk of Bias and GRADE Assessment

**Figure 2:**
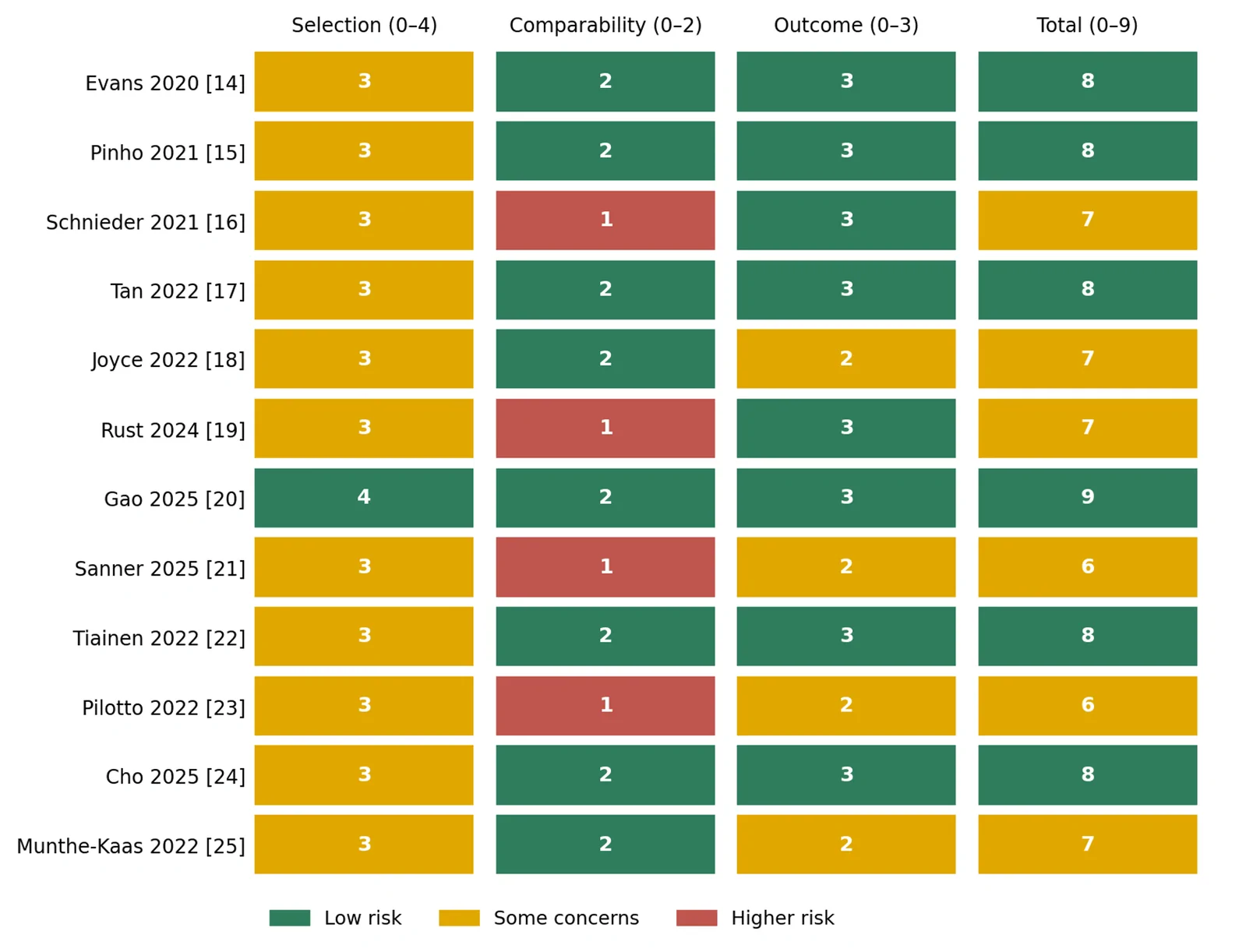
Newcastle-Ottawa Scale risk-of-bias summary across the three domains Studies included [14-25]

NOS appraisals are summarised in Table 3. Most cohorts drew consecutive or registry-based samples with objective outcome ascertainment (mortality via records/registries; functional outcome via the modified Rankin Scale (mRS)), supporting adequate selection and outcome domains. The principal threats to validity were the retrospective and, in some cases, single-centre study designs, as well as variable adjustment for baseline stroke severity, assessed using the National Institutes of Health Stroke Scale (NIHSS), and pre-stroke disability, which directly affect the comparability domain. Preliminary domain scores are summarised in Table 3 and visualised in Figure 2; all studies scored 6-9 of 9, indicating moderate-to-low risk of bias. These preliminary appraisals are to be confirmed by dual independent scoring.

**Table 3:** Risk-of-bias summary (Newcastle-Ottawa Scale)

Study	Selection (0-4)	Comparability (0-2)	Outcome (0-3)	Total (0-9)
Evans 2020 [14]	3	2	3	8
Pinho 2021 [15]	3	2	3	8
Schnieder 2021 [16]	3	1	3	7
Tan 2022 [17]	3	2	3	8
Joyce 2022 [18]	3	2	2	7
Rust 2024 [19]	3	1	3	7
Gao 2025 [20]	4	2	3	9
Sanner 2025 [21]	3	1	2	6
Tiainen 2022 [22]	3	2	3	8
Pilotto 2022 [23]	3	1	2	6
Cho 2025 [24]	3	2	3	8
Munthe-Kaas 2022 [25]	3	2	2	7

For mortality, evidence was upgraded from low to moderate based on a large, consistent effect (tripled risk) with dose-response gradient and corroboration across all frailty instruments (CFS, HFRS, laboratory-based frailty index (FI-LAB)). For functional outcome, certainty remains low due to substantial heterogeneity (I²=53%), differing mRS cut-offs (≥3 to ≥6), age thresholds (≥70 to ≥85), and wide CIs, though direction of effect is consistent. Narrative synthesis findings (per-unit analyses, longer-term outcomes) also rate low certainty, reflecting lack of formal pooling and reliance on individual observational studies (Table 4).

**Table 4:** GRADE summary GRADE: Grading of Recommendations Assessment, Development and Evaluation; HR: hazard ratio; CI: confidence interval; OR: odds ratio

Outcome	Studies	Effect estimate (95% CI)	Certainty	Reasoning
Mortality (early)	Pinho 2021 [15], Gao 2025 [20]	Pooled HR 3.24 (1.67-6.27)	⊕⊕⊕○ MODERATE	Large effect, no heterogeneity (I²=0%), consistent across instruments, but imprecision due to few studies
Poor functional outcome	Tan 2022 [17], Tiainen 2022 [22], Sanner 2025 [21]	Pooled OR 2.44 (1.11-5.35)	⊕⊕○○ LOW	Moderate heterogeneity (I²=53%), wide CIs, variable frailty thresholds and outcome definitions across cohorts

Frailty and Mortality

**Figure 3:**
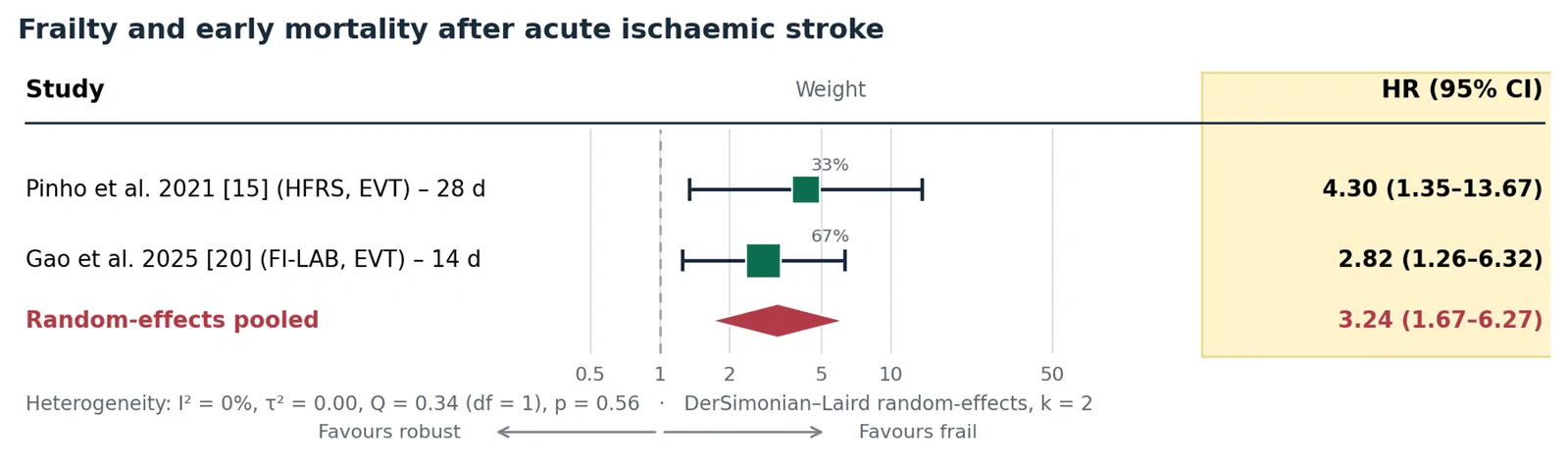
Random-effects meta-analysis (DerSimonian-Laird) of the adjusted association between frailty and early mortality after acute ischaemic stroke Studies included [15,20] EVT: endovascular therapy; HFRS: Hospital Frailty Risk Score; FI-LAB: laboratory-based Frailty Index

Every study reporting mortality found higher frailty associated with greater risk, across instruments and follow-up windows. In verified adjusted data, FI-LAB was an independent predictor of mortality after endovascular therapy in older large vessel occlusion (LVO)-AIS patients, with frail patients experiencing roughly threefold higher 12-month mortality than robust patients (adjusted HR 3.61, 95% CI 1.98-6.61) and elevated 14-day mortality (adjusted HR 2.82, 95% CI 1.26-6.32), and a clear dose-response across the frailty gradient [20]. Administratively derived frailty showed the same direction on a per-unit metric: each additional HFRS point independently predicted 90-day mortality after thrombectomy (adjusted OR 1.11, 95% CI 1.00-1.24) [16]. Two cohorts reported a dichotomous adjusted HR for early mortality and could be pooled: high frailty risk independently predicted 28-day mortality after endovascular therapy (adjusted HR 4.30, 95% CI 1.35-13.67) [15], and laboratory-index frailty predicted 14-day mortality (adjusted HR 2.82, 95% CI 1.26-6.32) [20]. A DerSimonian-Laird random-effects model gave a pooled adjusted HR of 3.24 (95% CI 1.67-6.27) with no detectable heterogeneity (I²=0%; Figure 3); however, this finding should be interpreted cautiously given that only two studies contributed to the pooled estimate. Longer-term and per-unit estimates pointed consistently the same way: the laboratory index also predicted 12-month mortality (adjusted HR 3.61, 95% CI 1.98-6.61) [20]; clinician-rated frailty (CFS ≥5) independently predicted one-year mortality (adjusted OR 3.86, 95% CI 1.48-10.05; per-point OR 1.71, 95% CI 1.19-2.45) [22]; each additional HFRS point independently predicted 90-day mortality (adjusted OR 1.11, 95% CI 1.00-1.24) [16]; and CFS independently predicted 28-day mortality after ischaemic stroke [14]. These are reported alongside, rather than combined with, the pooled early-mortality estimate because they mix hazard and ORs across different follow-up windows (Figure 3).

Frailty and Poor Functional Outcome

**Figure 4:**
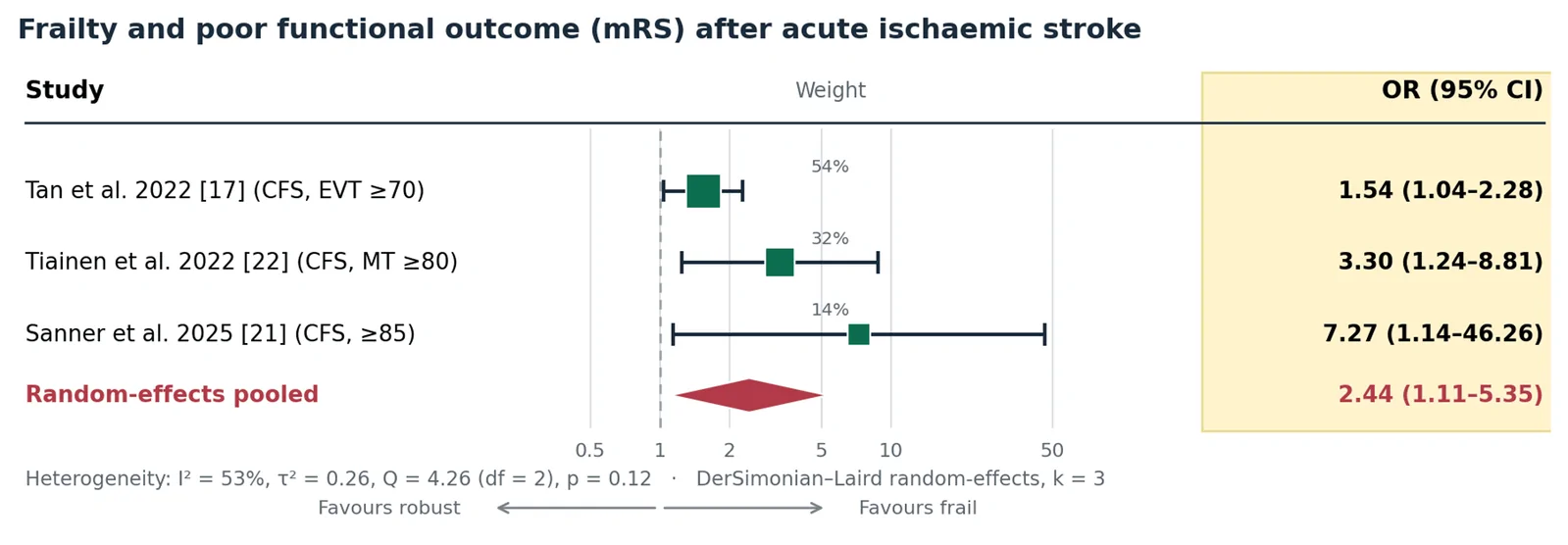
Random-effects meta-analysis (DerSimonian-Laird) of the adjusted association between frailty and poor functional outcome (dichotomised modified Rankin Scale) after acute ischaemic stroke in older adults Studies included [17,22,21] CFS: Clinical Frailty Scale; EVT: endovascular therapy; MT: mechanical thrombectomy

Frailty was consistently associated with worse functional outcome on the mRS, and three cohorts provided a dichotomous, maximally adjusted estimate suitable for pooling. In endovascular-therapy patients aged ≥70, frailty was associated with poorer 90-day functional outcome (adjusted OR 1.54, 95% CI 1.04-2.28) [17]; in thrombectomy-treated patients aged ≥80, frailty (CFS ≥5) more than tripled the odds of a poor three-month outcome (mRS 4-6; adjusted OR 3.30, 95% CI 1.24-8.81) [22]; and in patients aged ≥85, severe pre-stroke frailty (CFS ≥7) was independently associated with poor outcome (mRS ≥4; adjusted OR 7.27, 95% CI 1.14-46.26, and 11.42, 95% CI 1.59-81.88, in ischaemic stroke) [21]. A DerSimonian-Laird random-effects model yielded a pooled adjusted OR of 2.44 (95% CI 1.11-5.35), with moderate heterogeneity (I²=53%; Figure 2) attributable largely to differing frailty thresholds and case-mix. This estimate is closely concordant with the most recent published meta-analysis, which reported a pooled OR of 2.04 (95% CI 1.49-2.80) for poor functional outcome across stroke cohorts [26]. Consistent with this direction, each additional HFRS point independently predicted a worse 90-day mRS after thrombectomy (adjusted OR 1.13, 95% CI 1.00-1.27) [16], and index-based frailty predicted dependence across endovascular and mixed cohorts (Figure 4) [18,19].

Subgroup and Sensitivity Analyses

Pre-specified subgroup analyses by frailty instrument (CFS vs HFRS vs index-based) and by treatment context (reperfusion-treated vs mixed cohorts) were planned; however, due to an insufficient number of comparable estimates populating each stratum (fewer than two independent, metric-comparable studies per subgroup), these analyses could not be performed. Similarly, follow-up duration was considered as a potential source of heterogeneity, but formal subgroup analysis by timepoint was not feasible for the same reason. Assessment of publication bias using funnel-plot asymmetry and Egger's regression test was pre-specified but could not be undertaken, as the pooled analyses included fewer than 10 studies. These limitations are now clearly stated.

**Figure 5:**
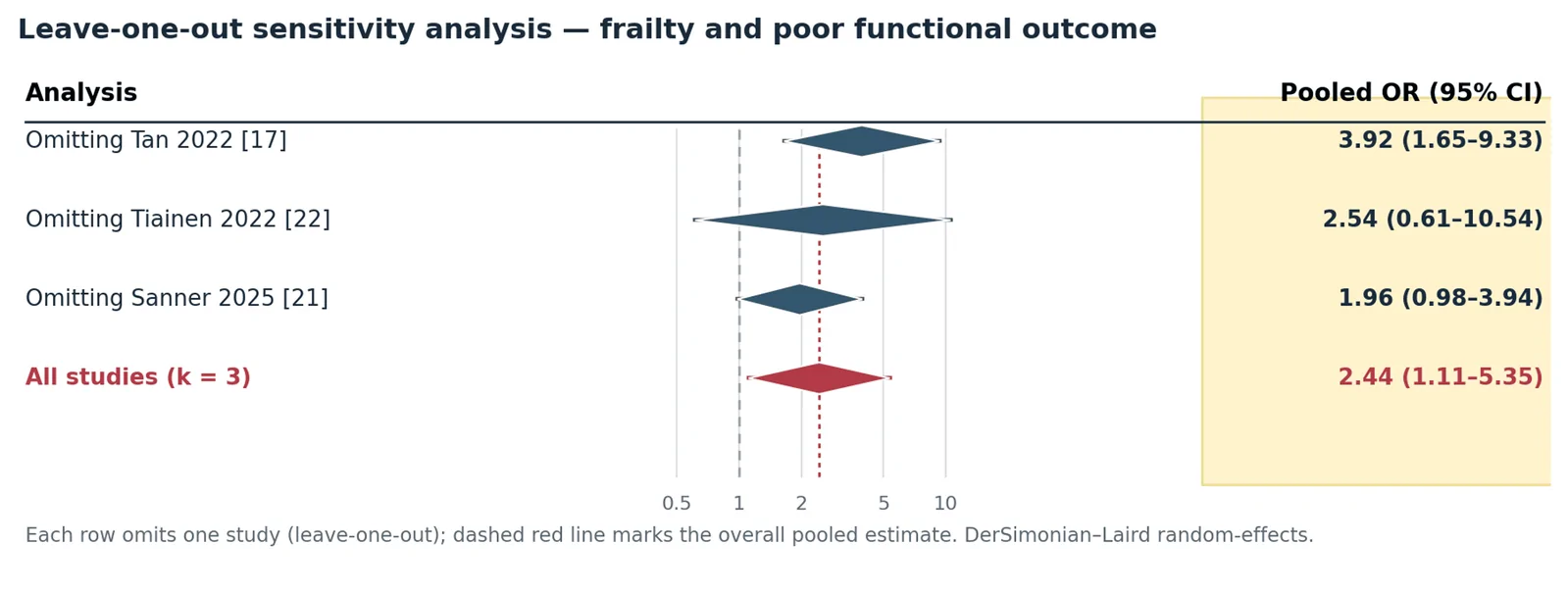
Leave-one-out sensitivity analysis of the random-effects meta-analysis for poor functional outcome; each row shows the pooled odds ratio recomputed with the indicated study removed, and the dashed red line marks the overall pooled estimate Studies included [17,22,21]

Because the functional-outcome pool comprised only three studies, its robustness was examined with a leave-one-out sensitivity analysis, recomputing the random-effects estimate with each study removed in turn (Figure 5). The pooled OR remained above 1.9 in every iteration (range 1.96 to 3.92 across the leave-one-out estimates, versus 2.44 with all three studies), indicating that no single cohort drove the association. In two of the three iterations, the 95% CI crossed unity; because each iteration leaves only two studies, this reflects the small number of contributing cohorts rather than a reversal of direction.

Discussion

This systematic review synthesises a coherent and rapidly maturing body of evidence indicating that frailty is a powerful predictor of clinical outcome after AIS in older adults, and that its prognostic signal is largely independent of the particular instrument used to capture it. Whether frailty was measured by the clinician-rated CFS, the administratively derived HFRS, a deficit-accumulation frailty index, or a FI-LAB, greater frailty was consistently associated with higher mortality and worse functional recovery. In the most fully verified dataset, laboratory-index frailty tripled 12-month mortality after endovascular therapy relative to robustness, with a graded relationship across the frailty spectrum [20]; per-unit HFRS increments independently predicted both 90-day mortality and poorer functional outcome after thrombectomy [16]; and clinician-rated frailty independently predicted early death after ischaemic stroke [14] and death or severe disability in the oldest old [21]. Convergent findings from CFS-based cohorts undergoing endovascular treatment [17], from deficit-index cohorts undergoing thrombectomy [18], and from blood-based frailty indices [19] reinforce the same conclusion. The consistency of direction across such methodologically diverse measures is itself an argument that the association reflects a real biological phenomenon - diminished physiological reserve - rather than an artefact of any single scale.

These results extend, rather than merely echo, the earlier literature. A previous systematic review established that frailty is common in acute stroke, affecting roughly a quarter of patients, and is broadly associated with adverse outcomes [9]; our synthesis sharpens that picture by focusing on older adults, separating mortality from functional endpoints, and foregrounding the treatment context that now dominates acute stroke care. A parallel synthesis reached similar conclusions about frailty and post-stroke outcomes across mixed populations [26], and a meta-analysis restricted to endovascular therapy likewise found frailty to be associated with worse outcomes after thrombectomy [27]. The laboratory-based frailty literature, summarised in a dedicated review, has repeatedly linked FI-LAB to mortality across clinical settings, lending external coherence to the stroke-specific findings reported here [28]. At the same time, outcome-prediction models developed for endovascular therapy have historically emphasised age, stroke severity, and imaging while largely omitting physiological reserve [29]; our findings suggest that incorporating a frailty term could improve the calibration of such models for the older patients who increasingly constitute the treated population.

Several mechanisms plausibly underlie the observed associations. Frailty reflects the accumulation of molecular, cellular, and organ-level deficits that erode the capacity to withstand an acute physiological insult; in the context of AIS, this reduced reserve translates into diminished tolerance of the ischaemic event itself, of reperfusion, and of the intensive care that acute stroke management entails [8]. Frail patients are more susceptible to post-stroke complications such as infection, delirium, and immobility-related events, each of which independently worsens survival and recovery. Frailty may also blunt neuroplasticity and reduce tolerance of, and adherence to, rehabilitation, thereby constraining the functional gains achievable after the acute phase. The laboratory-index findings add a mechanistic layer: a high burden of abnormal routine blood parameters - reflecting subclinical hepatic, renal, nutritional, inflammatory, and metabolic dysfunction - captures multisystem vulnerability that history- and function-based instruments may miss, and does so using data available before intervention, even in patients who cannot provide a pre-stroke history [20]. That some of the strongest signals emerge in reperfusion-treated cohorts is clinically salient, because it indicates that frailty conveys prognostic information over and above successful recanalisation.

The diversity of frailty instruments across the included studies is not merely a methodological nuisance but a practical guide to implementation. Clinician-rated scales such as the CFS are quick, inexpensive, and interpretable at the bedside, but they depend on a reliable account of pre-stroke function that is not always obtainable acutely, particularly for patients with impaired consciousness or without an accompanying informant [8,14]. Administrative indices such as the HFRS can be computed automatically from coded records and embedded in hospital information systems, enabling frailty ascertainment at scale, though they inherit the limitations of coding quality and were not originally designed for stroke-specific prognostication [5,16]. Laboratory-based indices occupy a complementary niche: because they are derived entirely from routine pre-procedural blood tests, they can be calculated objectively and rapidly even when a history cannot be taken - precisely the circumstance of many older patients presenting with LVO [6,20]. No single instrument is optimal across every setting; the pragmatic implication is that stroke services should select a frailty measure matched to their data environment and patient mix, while recognising that the prognostic conclusion is stable across the choice of tool.

The clinical implications warrant careful framing. The consistent, graded association between frailty and poor outcome argues for routine, structured frailty assessment at stroke presentation, using an instrument matched to the local setting-clinician-rated scales where a reliable pre-stroke history is obtainable, and administrative or laboratory indices where it is not. Such assessment can improve prognostic accuracy beyond age alone, support honest and individualised conversations with patients and families about likely trajectories, and identify candidates for proactive complication prevention, early rehabilitation planning, and, where appropriate, comprehensive geriatric co-management; recent stroke-specific reviews and a World Stroke Organization scientific statement similarly advocate embedding structured frailty assessment into stroke pathways [30,31]. Crucially, the evidence must not be read as a rationale for withholding reperfusion or other evidence-based therapies from frail patients. Frailty is a modifiable and partially reversible state, and prognostic pessimism risks becoming a self-fulfilling prophecy; the appropriate response is to individualise care and to test whether frailty-directed interventions - prehabilitation, nutritional optimisation, and structured geriatric management - can attenuate the excess risk, rather than to ration treatment on the basis of a frailty score [7]. The observation that frailty predicts outcome even among successfully treated patients is best understood as a call to broaden the therapeutic target beyond the occluded vessel to the whole patient.

A recurring theme across the reperfusion-treated cohorts is that frailty carries prognostic information beyond chronological age and beyond successful recanalisation, a finding with direct bearing on how older patients are selected for endovascular therapy [15,17,18,20]. Historically, advanced age has been used, implicitly or explicitly, to temper enthusiasm for aggressive treatment; yet the data assembled here suggest that biological reserve, not the calendar, is the more informative axis, echoing conceptual arguments that frailty should supplement age in stroke decision-making [8]. This matters because outcome-prediction models used to counsel patients and to design trials have generally omitted frailty [29], and because a meta-analysis confined to thrombectomy has already signalled that frailty tracks with worse post-procedural outcomes [27]. The correct inference is not that frail patients benefit less from reperfusion - the present evidence does not establish a differential treatment effect - but that frailty identifies a higher-risk group in whom baseline prognosis is worse and in whom peri-procedural vigilance, complication prevention, and realistic goal-setting are especially important. Disentangling prognostic value from predictive, treatment-modifying value is a priority for future work, ideally through individual-participant-data analyses of thrombectomy cohorts and, where feasible, frailty-stratified reporting within randomised trials.

This review has strengths. It concentrates on the older population in whom frailty is most prevalent and most consequential, distinguishes mortality from functional endpoints, prioritises maximally adjusted estimates, and applies a transparent synthesis strategy that pools only metric-comparable estimates and refrains from combining ORs with HRs or per-unit with dichotomous contrasts. Its principal contribution is to show that the frailty-outcome association in AIS is instrument-independent and robust across the treatment contexts that define contemporary care. By cataloguing which adjusted estimate each cohort reports and holding pooling to strict metric-comparability, the review also provides a reproducible scaffold that can be updated as further adjusted estimates are extracted and as new cohorts are published, and it makes explicit where the current evidence supports quantitative pooling and where it supports only structured narrative synthesis - an honesty that is itself useful for guideline developers weighing whether to recommend routine frailty assessment in acute stroke pathways.

Limitations

Our synthesis has important limitations. First, the included literature is clinically and methodologically heterogeneous: studies used different frailty instruments and thresholds, dichotomised the mRS at different cut-points, and reported outcomes at different timepoints, so that a proportion of the evidence is best represented as a structured narrative synthesis rather than a single pooled estimate, and pooled results should be interpreted alongside their I² values. Second, much of the strongest recent evidence derives from reperfusion-treated (endovascular) cohorts, which may limit generalisability to older patients managed conservatively. Third, most studies were observational, and several were retrospective and single-centre; residual confounding - particularly incomplete adjustment for baseline stroke severity and pre-stroke disability - cannot be excluded, and frailty and stroke severity are partially entangled. Fourth, ascertainment of frailty was frequently retrospective, and administrative indices depend on coding quality. Finally, publication and reporting biases are possible, and a formal assessment of small-study effects will be feasible only for outcomes to which a sufficient number of studies contribute. These considerations temper the certainty of the evidence, which we grade formally by outcome using GRADE.

## Conclusions

In older adults with AIS, frailty is a consistent and clinically meaningful predictor of mortality and poor functional outcome, and this association holds across clinician-rated, administrative, deficit-based, and laboratory-based instruments and across treatment contexts, including endovascular therapy. Frailty assessment should be integrated into routine acute stroke care to refine prognostication and individualise management - used to tailor treatment and support, not to deny it. Future prospective, multicentre studies with harmonised frailty instruments and outcome definitions, adequate adjustment for stroke severity, and evaluation of frailty-directed interventions are needed to convert this prognostic signal into improved outcomes.

## References

[1] GBD 2021 Stroke Risk Factor Collaborators (2024). Global, regional, and national burden of stroke and its risk factors, 1990-2021: a systematic analysis for the Global Burden of Disease Study 2021. Lancet Neurol.

[2] Hoogendijk EO, Afilalo J, Ensrud KE, Kowal P, Onder G, Fried LP (2019). Frailty: implications for clinical practice and public health. Lancet.

[3] Gordon EH, Hubbard RE (2022). Frailty: understanding the difference between age and ageing. Age Ageing.

[4] Mitnitski A, Song X, Rockwood K (2013). Assessing biological aging: the origin of deficit accumulation. Biogerontology.

[5] Gilbert T, Neuburger J, Kraindler J (2018). Development and validation of a Hospital Frailty Risk Score focusing on older people in acute care settings using electronic hospital records: an observational study. Lancet.

[6] Howlett SE, Rockwood MR, Mitnitski A, Rockwood K (2014). Standard laboratory tests to identify older adults at increased risk of death. BMC Med.

[7] O'Caoimh R, Sezgin D, O'Donovan MR, Molloy DW, Clegg A, Rockwood K, Liew A (2021). Prevalence of frailty in 62 countries across the world: a systematic review and meta-analysis of population-level studies. Age Ageing.

[8] Evans NR, Todd OM, Minhas JS (2022). Frailty and cerebrovascular disease: concepts and clinical implications for stroke medicine. Int J Stroke.

[9] Burton JK, Stewart J, Blair M, Oxley S, Wass A, Taylor-Rowan M, Quinn TJ (2022). Prevalence and implications of frailty in acute stroke: systematic review and meta-analysis. Age Ageing.

[10] Page MJ, McKenzie JE, Bossuyt PM (2021). The PRISMA 2020 statement: an updated guideline for reporting systematic reviews. BMJ.

[11] Wells GA, Shea B, O'Connell D (2021). The Newcastle-Ottawa Scale (NOS) for Assessing the Quality of Nonrandomised Studies in Meta-Analyses. https://www.ohri.ca/programs/clinical_epidemiology/oxford.asp.

[12] DerSimonian R, Laird N (1986). Meta-analysis in clinical trials. Control Clin Trials.

[13] Higgins JP, Thompson SG (2002). Quantifying heterogeneity in a meta-analysis. Stat Med.

[14] Evans NR, Wall J, To B, Wallis SJ, Romero-Ortuno R, Warburton EA (2020). Clinical frailty independently predicts early mortality after ischaemic stroke. Age Ageing.

[15] Pinho J, Küppers C, Nikoubashman O, Wiesmann M, Schulz JB, Reich A, Werner CJ (2021). Frailty is an outcome predictor in patients with acute ischemic stroke receiving endovascular treatment. Age Ageing.

[16] Schnieder M, Bähr M, Kirsch M (2021). Analysis of frailty in geriatric patients as a prognostic factor in endovascular treated patients with large vessel occlusion strokes. J Clin Med.

[17] Tan BY, Ho JS, Leow AS (2022). Effect of frailty on outcomes of endovascular treatment for acute ischaemic stroke in older patients. Age Ageing.

[18] Joyce N, Atkinson T, Mc Guire K (2022). Frailty and stroke thrombectomy outcomes-an observational cohort study. Age Ageing.

[19] Rust M, Küppers C, Nikoubashman O (2025). Blood-based frailty index in patients with acute ischemic stroke undergoing endovascular treatment. Cerebrovasc Dis.

[20] Gao W, Chen X, Annadurdyyev A, Cai L, Yu L, Xu Z, Zhu R (2025). Preprocedural frailty status and short- and long-term mortality risk after endovascular therapy in elderly acute ischemic stroke patients. J Nutr Health Aging.

[21] Sanner J, Thommessen B, von Euler M, Ström JO, Fure B (2025). Acute stroke in persons 85 years or older-clinical characteristics, impact of frailty, and predictors of outcome. Front Neurol.

[22] Tiainen M, Martinez-Majander N, Virtanen P, Räty S, Strbian D (2022). Clinical frailty and outcome after mechanical thrombectomy for stroke in patients aged ≥ 80 years. J Stroke Cerebrovasc Dis.

[23] Pilotto A, Brass C, Fassbender K (2022). Premorbid frailty predicts short- and long-term outcomes of reperfusion treatment in acute stroke. J Neurol.

[24] Cho TY, Hanger HC, Wilkinson TJ (2025). The association between frailty and stroke rehabilitation outcomes: a cohort study. Clin Rehabil.

[25] Munthe-Kaas R, Aam S, Saltvedt I (2022). Is Frailty Index a better predictor than pre-stroke modified Rankin Scale for neurocognitive outcomes 3-months post-stroke?. BMC Geriatr.

[26] Li J, Wan J, Wang H (2024). Role of frailty in predicting outcomes after stroke: a systematic review and meta-analysis. Front Psychiatry.

[27] Bao Q, Huang X, Wu X (2023). Implications of frailty in acute ischemic stroke receiving endovascular treatment: systematic review and meta-analysis. Aging Clin Exp Res.

[28] Sapp DG, Cormier BM, Rockwood K, Howlett SE, Heinze SS (2023). The frailty index based on laboratory test data as a tool to investigate the impact of frailty on health outcomes: a systematic review and meta-analysis. Age Ageing.

[29] Kremers F, Venema E, Duvekot M (2022). Outcome prediction models for endovascular treatment of ischemic stroke: systematic review and external validation. Stroke.

[30] Naeem F, Quinn T (2024). Frailty in stroke. Pract Neurol.

[31] Evans NR, Pinho J, Beishon L (2025). Frailty and stroke: global implications for assessment, research, and clinical care-a WSO scientific statement. Int J Stroke.

